# Supplementary material for: A stabilized MERS-CoV spike ferritin nanoparticle vaccine elicits robust and protective neutralizing antibody responses
Source: Nat Commun. 2026 Feb 5;17:1750. doi: 10.1038/s41467-026-68458-5 (PMC12913901; doi:10.1038/s41467-026-68458-5)
Supplement: Supplementary file 1 — Supplementary Information [file 41467_2026_68458_MOESM1_ESM.pdf]

Supplementary Information

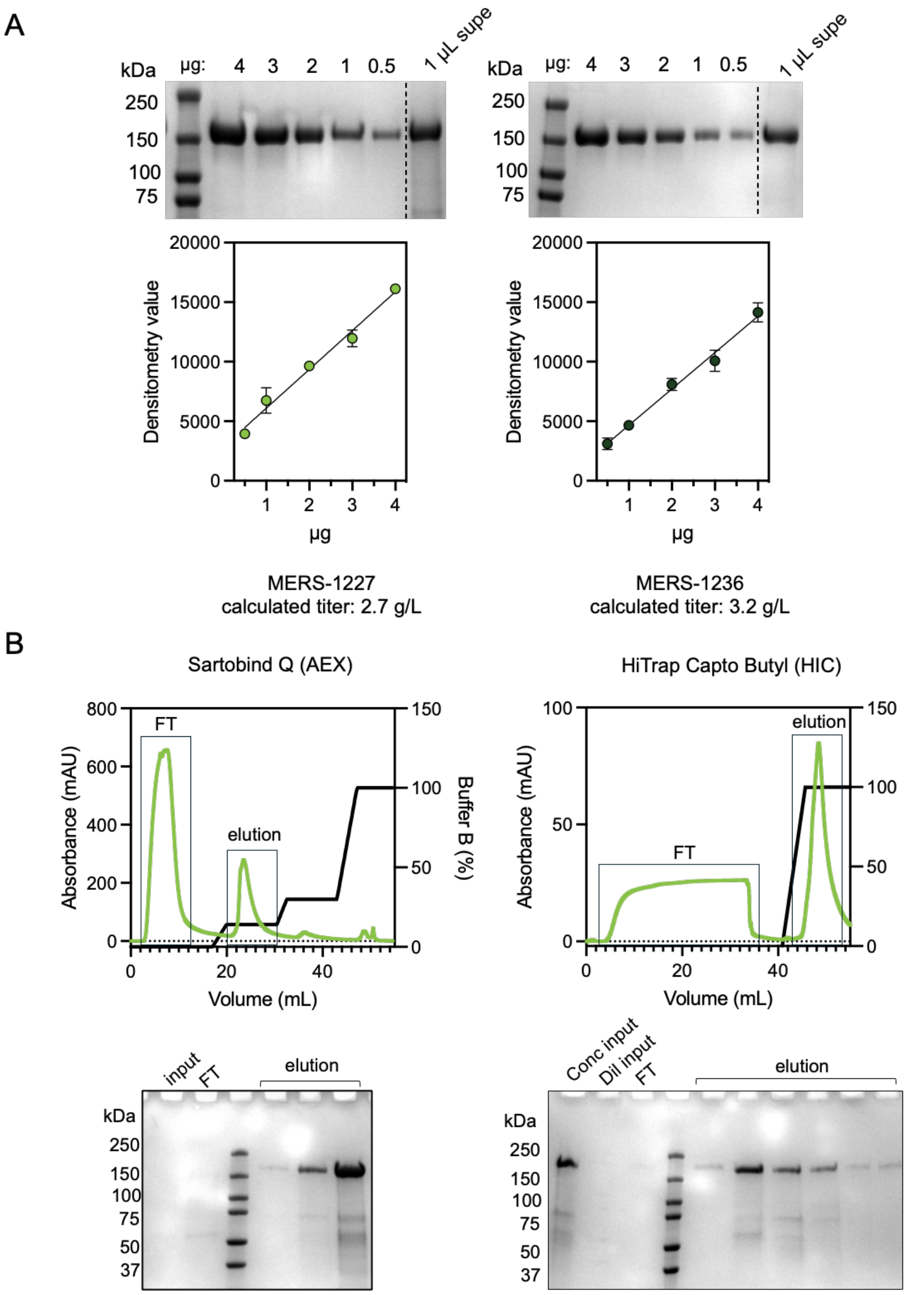

Figure S1. Quantitation of MERS FNPs in stable CHO pool supernatants and optimization of bind and elute chromatography purifications. (A) SDS-PAGE quantitation of MERS-1227 and MERS-1236 in 14-day fed-batch supernatants. Standard curves with 0.5-4 µg were generated using purified FNP proteins and quantified using densitometry. SDS-PAGE analysis was performed in duplicate; circles represent the mean and error bars represent the standard deviation. Densitometry was performed in ImageJ and quantitation was performed in GraphPad Prism. (B) Chromatograms obtained from AKTA purification on a Sartobind Q (left) and a HiTrap Capto Butyl (right) column. SDS-PAGE gels correspond to purification samples, with flow-through (FT) and elution fractions taken from the peaks boxed on the chromatograms above. Chromatography runs and SDS-PAGE analysis was conducted in duplicate. Source data are provided in the Source Data file.

A

B

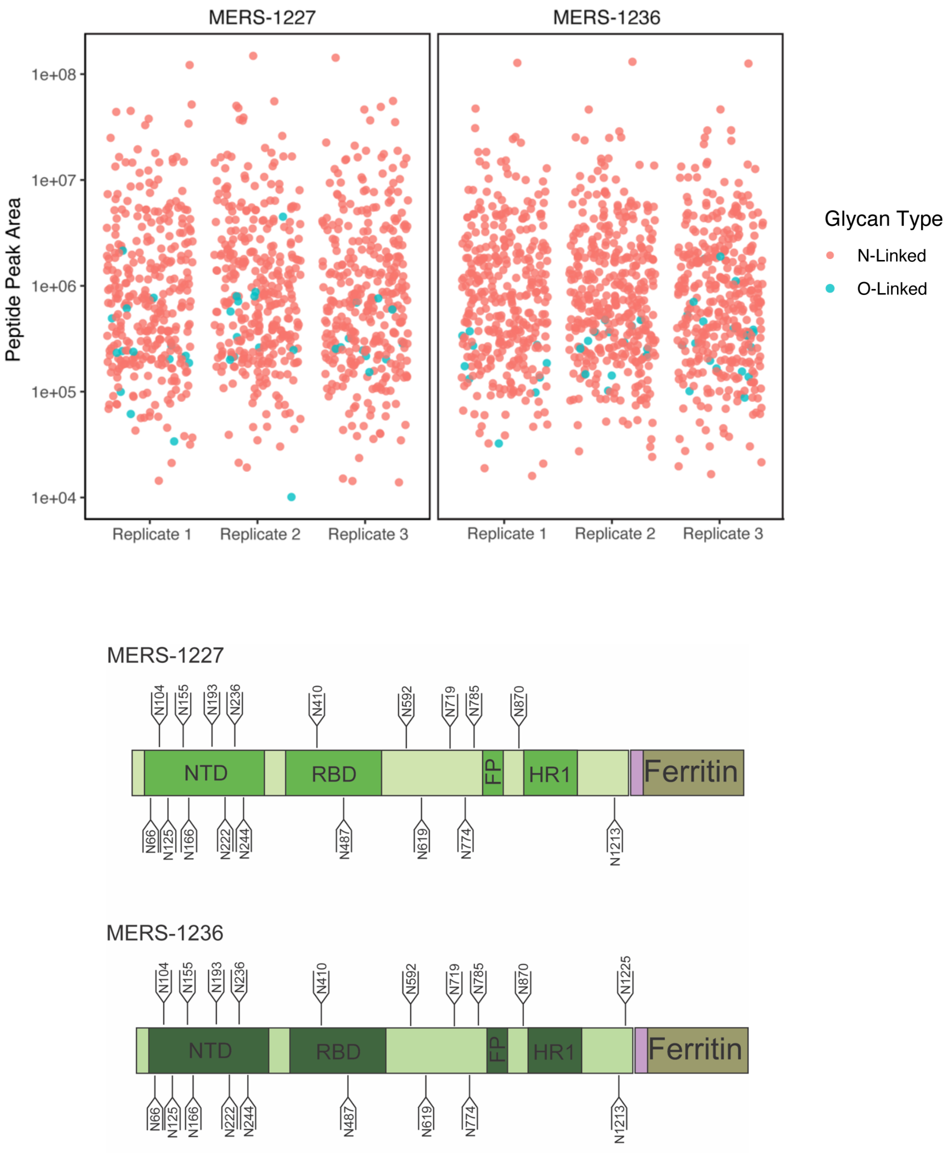

Figure S2. Characterization of MERS-1227 and MERS-1236 glycosylation using liquid chromatography-mass spectrometry (LC-MS/MS). (A) Semi-quantitative assessment of N-linked vs. O-linked glycans on MERS-1227 and MERS-1236 FNPs. Individual peptides were assigned to have N-linked or O-linked glycans and their precursor peak areas were integrated using PEAKS GlycanFinder software. Each dot represents the area of an individual peptide. Samples were analyzed in triplicate. (B) Primary structure of MERS-1227 and MERS-1236 annotated with positions of detected N-glycosites. Full data on the N-glycans at each site, detected O-glycans, and summarized peak areas for each replicate are reported in Supplementary Data 1.

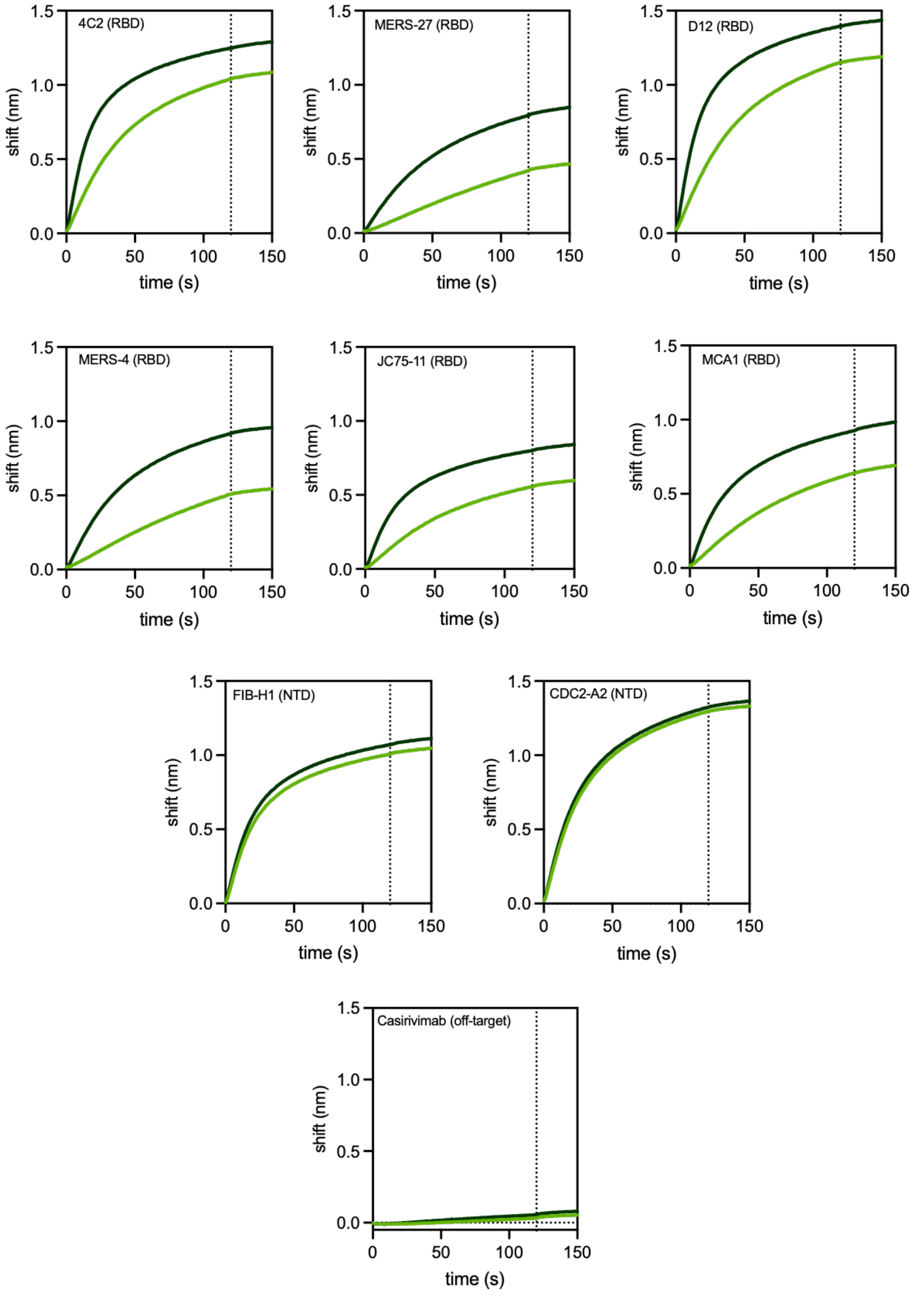

Figure S3. BLI binding of a panel of MERS-CoV mAbs to MERS-1227 and MERS-1236. mAbs were loaded onto anti-human capture sensor tips and dipped into either MERS-1227 or MERS-1236 wells at 100 µg/mL FNP. Dashed line indicates the end of the association step. Binding assays were conducted in duplicate and the mean value of the replicates is shown. Source data are provided in the Source Data file.

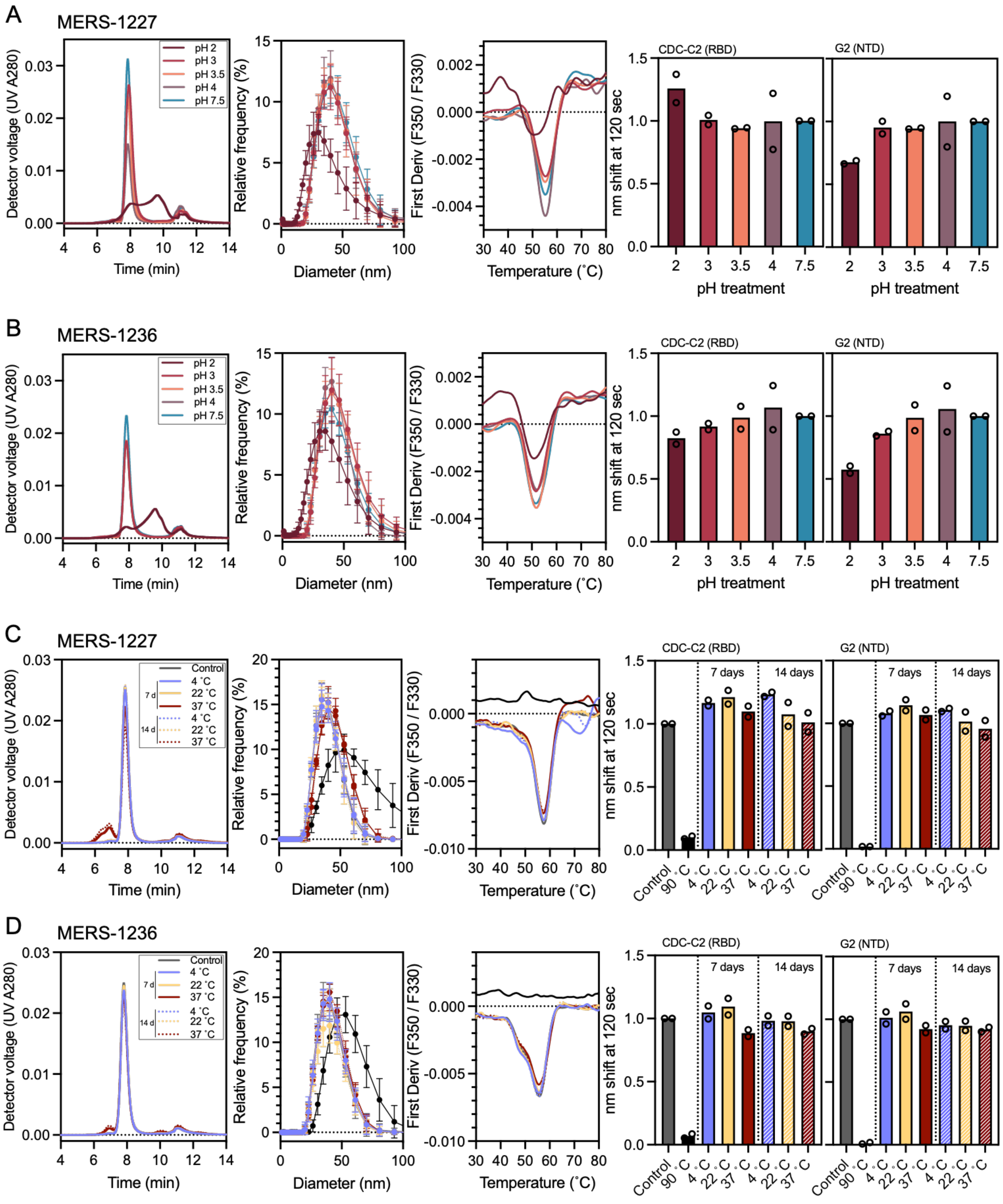

A
MERS-1227
pH 2
pH 3
pH 3.5
pH 4
pH 7.5
Detector voltage (UV A280)
Time (min)
Relative frequency (%)
Diameter (nm)
First Deriv (F350 / F330)
Temperature (˚C)
CDC-C2 (RBD)
G2 (NTD)
nm shift at 120 sec
pH treatment
B
MERS-1236
C
MERS-1227
Control
7 d
14 d
4 ˚C
22 ˚C
37 ˚C
90 ˚C
7 days
14 days
D
MERS-1236

Figure S4. MERS-1227 and MERS-1236 retain nanoparticle structure and antigenicity following low-pH treatment and extended incubation up to 37 ˚C. (A) Biophysical characterization of MERS-1227 following pH treatment at pH 2, 3, 3.5, 4, and 7.5. Panels show analytical SEC (left), DLS (middle left), DSF (middle right), and BLI (right two panels) with an RBD-targeting mAb, CDC-C2, and an NTD-targeting antibody, G2. pH treatments were conducted in duplicate and each sample was then characterized using each assay. Curves shown for SEC, DLS, and DSF represent one replicate. DLS measurements are taken via 10 individual acquisitions from a single technical replicate; points represent the mean value and error bars represent the standard deviation of the individual acquisitions, with one representative replicate shown. BLI plots show the mean nm shift value of the two treatment replicates at 120 s normalized to the pH 7.5 control, and error bars represent SD. (B) Biophysical characterization of MERS-1236 following pH treatment as described in (A). (C) Biophysical characterization of MERS-1227 following incubation at 4, 22, or 37 ˚C for 7 and 14 days. Solid lines represent 7-day treated samples and dashed lines represent 14-day treated samples. Control samples include a sample not subjected to any incubation (gray) and a sample heat-treated at 90 ˚C for 30 min (black). The 90 ˚C heat-treated sample was included in the DLS, DSF, and BLI analyses but was excluded from SEC analysis. BLI measurements were normalized to the untreated control sample. (D) Biophysical characterization of MERS-1236 following thermal incubations as described in (C). Source data are provided in the Source Data file.

A

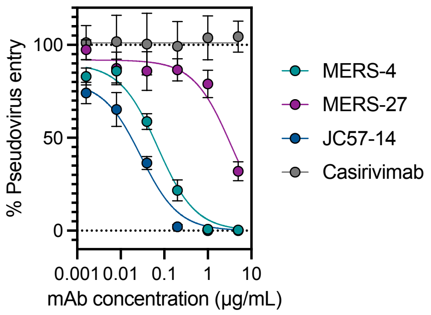

B

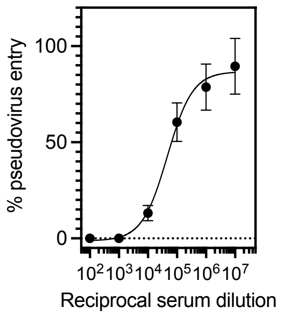

mAb	Determined IC_{50} (µg/mL)	Literature IC_{50} (µg/mL)
MERS-4	0.071	0.056 [55]
MERS-27	3.4	9.6 [55]
JC57-14	0.029	0.0084 [13]

Assay	Serum dilution
Live-virus ($PRNT_{90}$)	320 [51]
Pseudotyped lentivirus (IC_{90})	6810

Figure S5. Validation of MERS-CoV spike-pseudotyped lentivirus neutralization assay using monoclonal antibodies and convalescent camel serum. (A) Pseudovirus neutralization assays run with serial dilutions of known MERS-CoV spike-targeting mAbs. A SARS-CoV-2 spike-targeting mAb (casirivimab) was included as a negative control. Samples were run in quadruplicate; circles represent the average % pseudovirus entry at each mAb concentration, and error bars represent the standard deviation. (B) Pseudovirus neutralization run with serum obtained from a camel 42 days following MERS-CoV challenge. Sample was run in n=10 replicates; circles represent the average % pseudovirus entry at each serum concentration, and error bars represent the standard deviation. Source data are provided in the Source Data file.

Serum pool	$PRNT_{90}$	$PRNT_{80}$	$PRNT_{50}$
MERS-1227 Day 0	<10	<10	40
MERS-1227 – Day 42 (10 μg dose)	160	160	≥320
MERS-1236 – Day 0	<10	<10	<10
MERS-1236 – Day 42 (10 μg dose)	≥ 320	≥320	≥320

Table S1. Pooled mouse serum from MERS-1227 and MERS-1236 immunizations neutralizes live MERS-CoV EMC/2012 via plaque reduction neutralization assay. Mouse serum taken at day 0 or 42 from the immunization shown in Figure 4 was pooled (40 μL per mouse) and evaluated using a live-virus PRNT assay in a single replicate. Seropositive and seronegative camel sera was used as assay controls. Serum dilutions corresponding to $PRNT_{90}$, $PRNT_{80}$, and $PRNT_{50}$ are shown for each pooled serum sample.

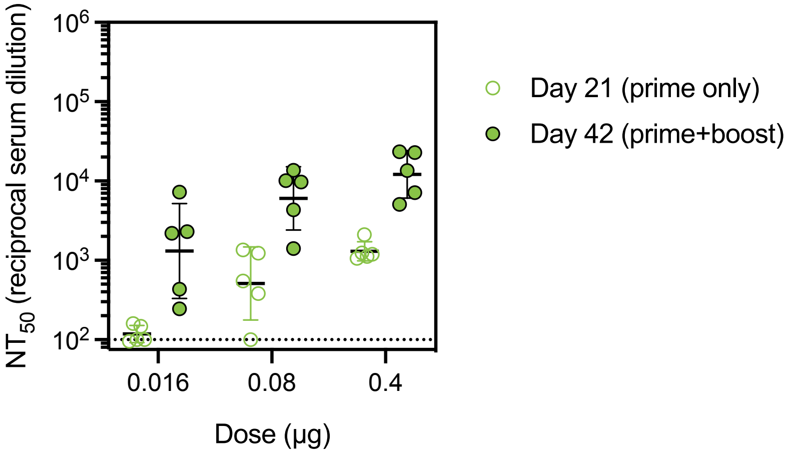

Figure S6. Dose de-escalation of MERS-1227 adjuvanted with Alhydrogel reveals quantifiable pseudovirus neutralizing titers following two doses as low as 0.016 µg FNP protein. Mice (n = 5) were immunized with 0.016, 0.08, or 0.4 µg MERS-1227 FNP protein adjuvanted with 150 µg Alhydrogel at days 0 and 21. Serum was collected at day 21 (prime only) and 42 (prime + boost) and assayed using pseudovirus neutralization. Each circle represents the NT_{50} for an individual mouse from quadruplicate measurements, bars represent the GMT, and error bars represent the geometric mean SD. Source data are provided in the Source Data file.

A

Identifier	Birthdate	Weight (kg) at time of first dose	Antigen
NV1702	15-Nov-17	5.22	MERS-1227
BC2312	26-Aug-18	4.54	MERS-1227
BC2483	30-Aug-18	4.5	MERS-1236
FR2573	6-Mar-18	6.14	MERS-1236
FR2864	2-Sep-18	4.56	MERS-1236
FR2913	8-Jul-18	6.02	MERS-1227
FR2923	9-Jul-18	4.36	MERS-1227
FR3151	27-Sep-18	4.4	MERS-1227
FR3188	29-Jul-18	4.16	MERS-1236
FR3267	3-Jan-18	4.37	MERS-1236

B

Identifier	Age (years)	Vaccine Group	Antigen and dose
0892	6	1	20 µg MERS-1227
3529	7	1	20 µg MERS-1227
0890	8	1	20 µg MERS-1227
0897	7	1	20 µg MERS-1227
3526	9	1	20 µg MERS-1227
3523	5	2	200 µg MERS-1227
0895	7	2	200 µg MERS-1227
0879	9	2	200 µg MERS-1227
3528	7	2	200 µg MERS-1227
3527	9	2	200 µg MERS-1227
0893	6	3	20 µg MERS-1236
0896	7	3	20 µg MERS-1236
0898	9	3	20 µg MERS-1236
6532	7	3	20 µg MERS-1236
0891	7	3	20 µg MERS-1236
3525	4	4	200 µg MERS-1236
3524	8	4	200 µg MERS-1236
3522	7	4	200 µg MERS-1236
0889	7	4	200 µg MERS-1236
0894	9	4	200 µg MERS-1236
6530	6	5	Placebo
3521	7	5	Placebo
6533	7	5	Placebo
6531	7	5	Placebo
0880	9	5	Placebo

Table S2. Age and vaccine group for NHPs and alpacas included in immunization studies. (A) Ages and weights (kg) of NHPs immunized with MERS-1227 and MERS-1236 FNP proteins. (B) Ages and vaccine group designations for alpacas used in alpaca MERS-CoV challenge study.

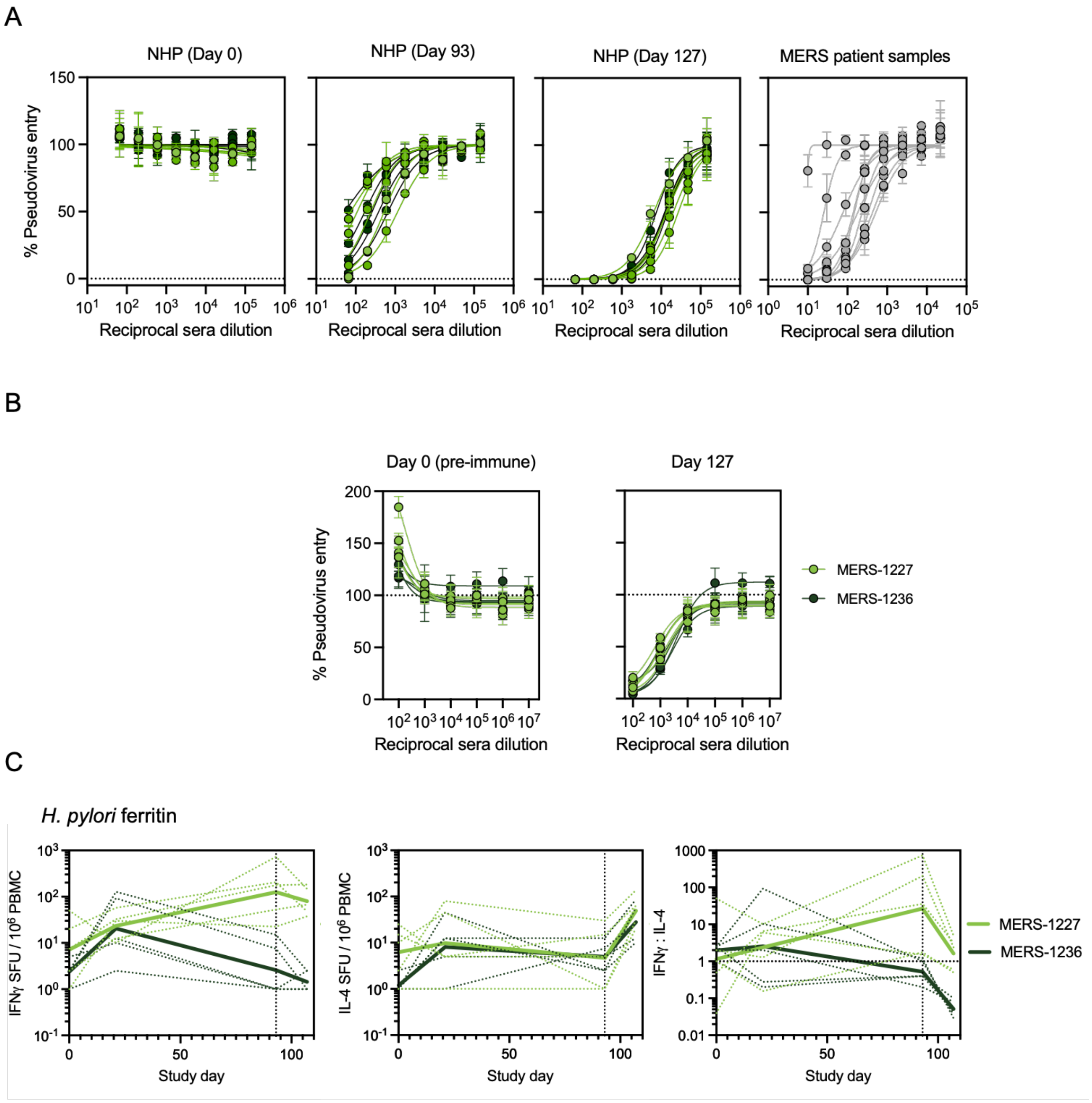

Figure S7. Characterization of NHP immunogenicity following MERS-1227 and MERS-1236 immunization. (A) Dose-response curves from VSV pseudovirus assay with day 0, 93, and 127 serum from immunized NHPs and MERS patient serum samples. Samples were assessed with a 3-fold dilution series, with NHP sera staring at a 1:66 dilution and MERS patient serum starting at a 1:10 dilution. Each point represents the mean % pseudovirus entry taken from two biological replicates each performed in duplicate. Error bars represent standard deviation. (B) Dose-response curves from MjHKU4r-CoV-1 pseudovirus neutralization assay with day 0 (pre-immune) and day 127 serum from immunized NHPs. Serum was assessed starting at a 1:100 dilution with 10-fold dilution steps. Each point represents the mean % pseudovirus entry from quadruplicate measurements for a single NHP at each serum concentration and error bars represent standard deviation. (C) IFNγ (left) and IL-4 (right) responses to *H. pylori* ferritin quantified via ELISpot. The solid lines represent the average values of each antigen group and the dashed lines represent the GMTs by individual NHP. The reported values for each individual NHP were obtained using the median of two technical replicates. Source data are provided in the Source Data file.

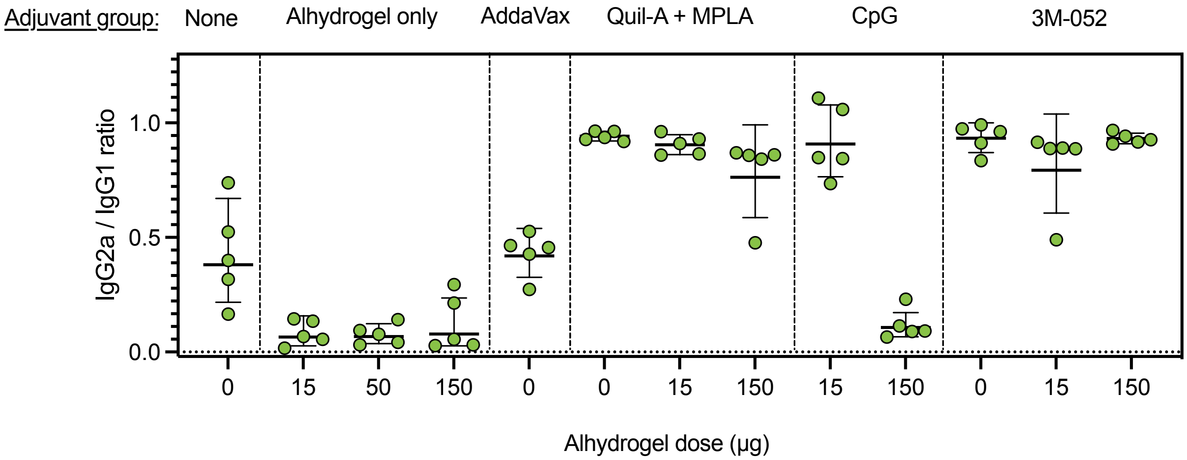

Figure S8. Ratio of IgG2a and IgG1 responses to MERS-CoV spike determined by Luminex reveal Th1/Th2 skewing effects of various adjuvants in BALB/c mice. Spike-specific IgG2a and IgG1 levels were determined using a Luminex binding assay for each mouse following two immunizations (day 42) with MERS-1227 formulated with adjuvant compositions indicated at the top. Binding was performed in duplicate using biotinylated MERS-CoV trimer bound to streptavidin Luminex beads. The ratio was obtained by dividing the mean IgG2a value by the mean IgG1 value. The circles represent the ratio for each mouse, bars represent the geometric mean for each group, and error bars represent the geometric SD. Source data are provided in the Source Data file.

A

mRNA	Encapsulation efficiency (%)	Size (nm)	PDI	Zeta potential (mV)
MERS-1227 ferritin	96.3	102.8	0.1259	-1.138
MERS-1227 cell-anchored	96.4	105.6	0.1169	-4.734
MERS-FL cell-anchored	96.9	104.3	0.02754	-4.252

B

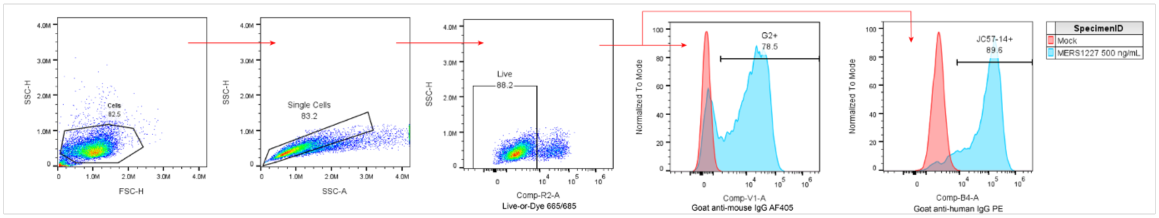

C

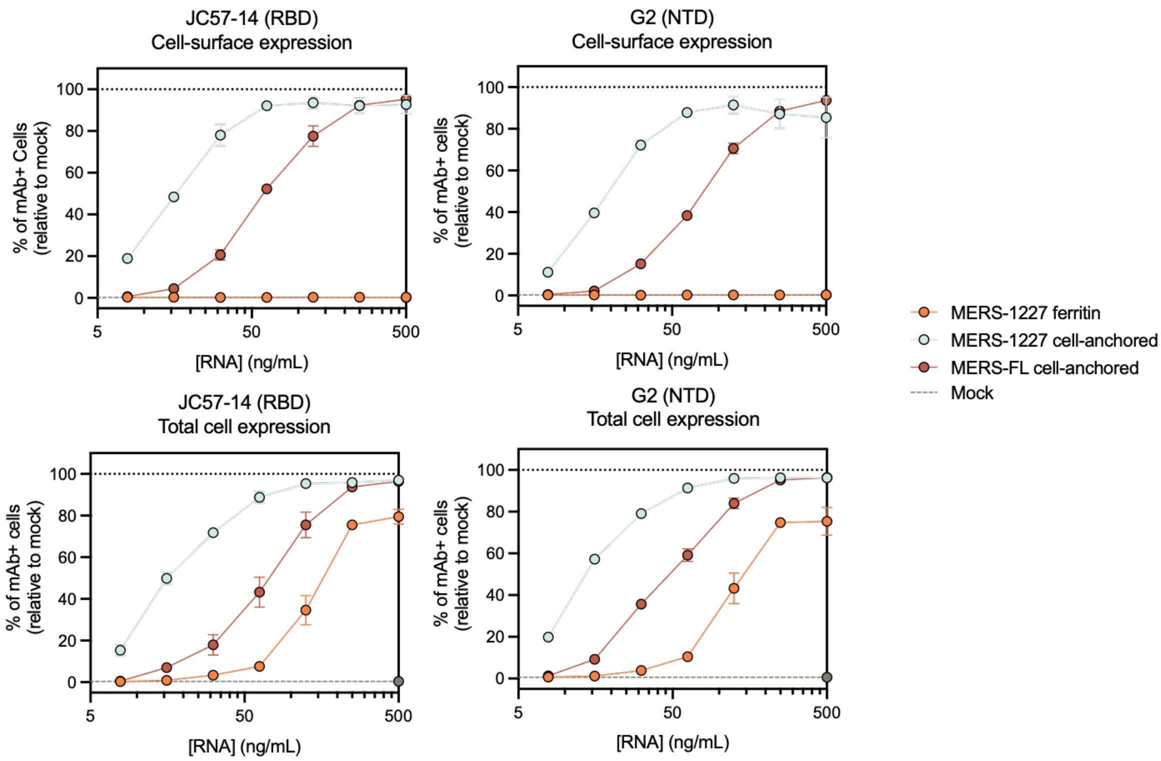

Figure S9. mRNA/LNP analytical characterization and assessment of cellular potency. (A) Analytical characterization of mRNA/LNP complexes including mRNA encapsulation efficiency, size as determined by dynamic light scattering (DLS), polydispersity index, and zeta potential. (B) Cellular expression of MERS-CoV antigens encoded in mRNAs was evaluated by transfecting HeLa cells in duplicate with mRNA/LNP complexes. Cells were left untreated (top panels) or fixed and permeabilized (bottom panels) and stained with either a MERS-CoV RBD (JC57-14) or NTD (G2) antibody and assayed using flow cytometry. A representative gating strategy is shown. (C) Antibody binding determined using the gating strategy from (B) is plotted as a function of LNP concentration. Circles present the mean % positive cells from duplicate cell wells and error bars represent standard deviation. Source data are provided in the Source Data file.

Identifier	Homozygous or heterozygous (HO/HZ)	Group	Dose (µg)	Day of euthanasia or death	Lung histopathology			PFU / gram lung
					Necrosis	Inflammation	Vascular changes	
34	HZ	1	0.016	3	0	0	0	<100
40	HO	1	0.016	3	0	1	0	9.00E+02
49	HZ	1	0.016	3	0	0	0	1.40E+04
56	HZ	1	0.016	3	0	0	0	<100
64	HO	1	0.016	7	0	0	0	ND
74	HZ	1	0.016	7	0	1	1	ND
80	HZ	1	0.016	10	0	0	0	ND
87	HZ	1	0.016	7	0	0	0	ND
35	HO	2	0.4	3	0	0	0	<100
41	HZ	2	0.4	3	0	1	0	<100
50	HO	2	0.4	3	0	1	1	<100
57	HZ	2	0.4	3	ND	ND	ND	<100
68	HZ	2	0.4	10	0	0	0	ND
75	HZ	2	0.4	10	0	0	0	ND
81	HZ	2	0.4	8 (pregnant)	0	0	0	ND
88	HO	2	0.4	10	0	0	0	ND
25	HO	5	0	3	0	0	0	<100
39	HZ	5	0	3	0	0	0	1.80E+03
48	HO	5	0	3	0	1	0	2.00E+03
55	HZ	5	0	3	0	0	0	1.10E+03
63	HZ	5	0	7	0	1	0	ND
79	HZ	5	0	9	0	0	0	ND
84	HO	5	0	8	0	0	0	ND

Table S3. Characteristics of mice and histopathology lung scoring from hDPP4 mouse challenge study. hDDP4 status (homozygous or heterozygous), MERS-1227 dose, and outcome for hDPP4 mice in MERS-CoV challenge study. Lung histopathology scoring as follows: 0 = no apparent changes (with normal limits = wnl); 1 = minimal change; 2 = mild change; 3 = moderate change; 4 = severe change in <50% of section; 5 = severe change in > 50% of section. Viral load in the lung tissue was determined for animals sacrificed at day 3 post challenge and is expressed as PFU / gram lung tissue. ND = not determined.

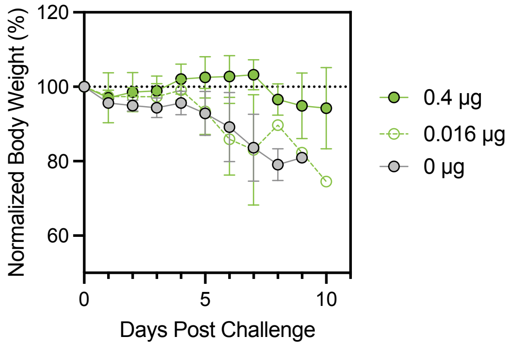

Figure S10. Weight loss following MERS-CoV challenge in hDPP4 mice immunized with MERS-1227. Mice (n = 4 for 0.4 µg and 0.016 µg dose groups and n = 3 for 0 µg dose group) were immunized with 0, 0.016, or 0.4 µg MERS-1227 FNP protein adjuvanted with 150 µg Alhydrogel at days 0 and 21 and challenged with EMC/2012 MERS-CoV at day 42. Mice were weighed daily post-challenge and weights are plotted as the averaged % body weight as compared to day 0 post-challenge. Each circle represents the mean weight per group at each timepoint and error bars represent SD. Mice that died were excluded from each time-point. Source data are provided in the Source Data file.
