# Supplementary material for: A stabilized MERS-CoV spike ferritin nanoparticle vaccine elicits robust and protective neutralizing antibody responses
Source: Nat Commun. 2026 Feb 5;17:1750. doi: 10.1038/s41467-026-68458-5 (PMC12913901; doi:10.1038/s41467-026-68458-5)
Supplement: Supplementary file 3 — Description of Additional Supplementary Files [file 41467_2026_68458_MOESM3_ESM.pdf]

Description of Additional Supplementary Files

File Name: Supplementary Data 1
Description: **Glycan profiles of MERS-1227 and MERS-1236 FNP proteins.** Following tryptic digestions, peptides were analyzed by LC-MS/MS, and glycans were identified using PEAKS 11 GlycanFinder software. Identified glycan compositions at each glycosylated amino acid site are reported. Peak areas for each replicate reflect the extracted ion peak areas as determined by automatic integration by GlycanFinder. Entries with missing peak areas were not integrable, suggesting low abundance of these species. For relative quantitation of the glycan compositions, the relative % ion abundance for a given glycan composition, normalized to the total glycan ion abundance for that site, was calculated. Only glycans with quantifiable peak areas in at least 2 of the three replicates were considered for this quantitative estimate. Owing to potentially large differences in the ionization efficiency of glycosylated vs. nonglycosylated peptides, we did not consider non-glycosylated peptides in this analysis nor did we attempt to estimate overall glycosite occupancy from these data. The raw data are deposited to PRIDE with the dataset identifier PXD059802.

File Name: Supplementary Data 2
Description: **Sequences of antibodies, recombinant proteins, and pseudovirus MERS-CoV spikes.**
