# Supplementary material for: A stabilized MERS-CoV spike ferritin nanoparticle vaccine elicits robust and protective neutralizing antibody responses
Source: Nat Commun. 2026 Feb 5;17:1750. doi: 10.1038/s41467-026-68458-5 (PMC12913901; doi:10.1038/s41467-026-68458-5)
Supplement: Supplementary file 5 — Reporting Summary [file 41467_2026_68458_MOESM5_ESM.pdf]

Corresponding author(s): Abigail Powell
Last updated by author(s): Dec 3, 2025

Reporting Summary

Nature Portfolio wishes to improve the reproducibility of the work that we publish. This form provides structure for consistency and transparency in reporting. For further information on Nature Portfolio policies, see our Editorial Policies and the Editorial Policy Checklist.

Statistics

For all statistical analyses, confirm that the following items are present in the figure legend, table legend, main text, or Methods section.

n/a	Confirmed	
☐	☒	The exact sample size (*n*) for each experimental group/condition, given as a discrete number and unit of measurement
☐	☒	A statement on whether measurements were taken from distinct samples or whether the same sample was measured repeatedly
☐	☒	The statistical test(s) used AND whether they are one- or two-sided<br>*Only common tests should be described solely by name; describe more complex techniques in the Methods section.*
☒	☐	A description of all covariates tested
☐	☒	A description of any assumptions or corrections, such as tests of normality and adjustment for multiple comparisons
☐	☒	A full description of the statistical parameters including central tendency (e.g. means) or other basic estimates (e.g. regression coefficient) AND variation (e.g. standard deviation) or associated estimates of uncertainty (e.g. confidence intervals)
☐	☒	For null hypothesis testing, the test statistic (e.g. *F*, *t*, *r*) with confidence intervals, effect sizes, degrees of freedom and *P* value noted<br>*Give P values as exact values whenever suitable.*
☒	☐	For Bayesian analysis, information on the choice of priors and Markov chain Monte Carlo settings
☒	☐	For hierarchical and complex designs, identification of the appropriate level for tests and full reporting of outcomes
☒	☐	Estimates of effect sizes (e.g. Cohen's *d*, Pearson's *r*), indicating how they were calculated

Our web collection on statistics for biologists contains articles on many of the points above.

Software and code

Policy information about availability of computer code

Data collection

SDS-PAGE: iBright CL750 1.8.0
SEC-MALS: Empower 7.3.0 and ASTRA 8.3.0.132
DLS/DSF: PR.Panta Control v1.9
BLI: Octet BLI Discovery 13.0.1.19
nsTEM: SerialEM 4.2.4
Lentivirus neutralization: BioTek Gen5 3.13.15
Luminex: Luminex INTELLIFLEX Bundle - 2.1.1015
AKTA FPLC: UNICORN 7.8 (Build 7.8.0.2159)
LC/MS: Thermo Scientific Xcalibur version 4.7
Flow cytometry: Cytek SpectroFlo
ELISpot:KS ELISPOT software v4.10

Data analysis	SEC-MALS: ASTRA 8.3.0.132<br>DLS/DSF: PR.Panta Analysis v1.9<br>nsTEM: RELION 5.0.0<br>BLI: Octet Analysis Studio 13.0.1.35<br>LC/MS: PEAKS Studio 11, ggplot2 3.5.0, R version 4.3.3<br>GraphPad Prism 10.5.0<br>Flow cytometry: FlowJo version 11<br>ImageJ 1.35t

For manuscripts utilizing custom algorithms or software that are central to the research but not yet described in published literature, software must be made available to editors and reviewers. We strongly encourage code deposition in a community repository (e.g. GitHub). See the Nature Portfolio guidelines for submitting code & software for further information.

Data

Policy information about availability of data

All manuscripts must include a data availability statement. This statement should provide the following information, where applicable:

- Accession codes, unique identifiers, or web links for publicly available datasets
- A description of any restrictions on data availability
- For clinical datasets or third party data, please ensure that the statement adheres to our policy

Data supporting the findings of this study are available from the manuscript and the supplemental information. The mass spectrometry proteomics data have been deposited to the ProteomeXchange Consortium via the PRIDE99 partner repository with the dataset identifier PXD059802. All source data are provided with this paper in the Source Data file. PDB structures used to generate Figure 1B are available via accession codes 7X27 [http://doi.org/10.2210/pdb7X27/pdb] and 6XCM [http://doi.org/10.2210/pdb6XCM/pdb].

Research involving human participants, their data, or biological material

Policy information about studies with human participants or human data. See also policy information about sex, gender (identity/presentation), and sexual orientation and race, ethnicity and racism.

Reporting on sex and gender	*Use the terms sex (biological attribute) and gender (shaped by social and cultural circumstances) carefully in order to avoid confusing both terms. Indicate if findings apply to only one sex or gender; describe whether sex and gender were considered in study design; whether sex and/or gender was determined based on self-reporting or assigned and methods used.*<br>*Provide in the source data disaggregated sex and gender data, where this information has been collected, and if consent has been obtained for sharing of individual-level data; provide overall numbers in this Reporting Summary. Please state if this information has not been collected.*<br>*Report sex- and gender-based analyses where performed, justify reasons for lack of sex- and gender-based analysis.*
Reporting on race, ethnicity, or other socially relevant groupings	*Please specify the socially constructed or socially relevant categorization variable(s) used in your manuscript and explain why they were used. Please note that such variables should not be used as proxies for other socially constructed/relevant variables (for example, race or ethnicity should not be used as a proxy for socioeconomic status).*<br>*Provide clear definitions of the relevant terms used, how they were provided (by the participants/respondents, the researchers, or third parties), and the method(s) used to classify people into the different categories (e.g. self-report, census or administrative data, social media data, etc.)*<br>*Please provide details about how you controlled for confounding variables in your analyses.*
Population characteristics	*Describe the covariate-relevant population characteristics of the human research participants (e.g. age, genotypic information, past and current diagnosis and treatment categories). If you filled out the behavioural & social sciences study design questions and have nothing to add here, write "See above."*
Recruitment	*Describe how participants were recruited. Outline any potential self-selection bias or other biases that may be present and how these are likely to impact results.*
Ethics oversight	*Identify the organization(s) that approved the study protocol.*

Note that full information on the approval of the study protocol must also be provided in the manuscript.

Field-specific reporting

Please select the one below that is the best fit for your research. If you are not sure, read the appropriate sections before making your selection.

☒ Life sciences ☐ Behavioural & social sciences ☐ Ecological, evolutionary & environmental sciences

For a reference copy of the document with all sections, see nature.com/documents/nr-reporting-summary-flat.pdf

Life sciences study design

All studies must disclose on these points even when the disclosure is negative.

Sample size	No sample size calculations were conducted prior to animal studies. Studies were designed to be descriptive in nature and evaluate qualitative

	differences in outcome.
Data exclusions	One dose group (0.4 µg) was omitted from our mouse immunogenicity study because we did not conduct this dose level for all three antigens evaluated in the study. To improve clarity of interpretation across the three antigens, we chose to present only the doses (2 µg and 10 µg) that had been tested with all three groups.
Replication	No additional study replicates were conducted aside from the presented data.
Randomization	Wild-type BALB/c mice did not have specified criteria for randomization in experimental groups. NHPs and alpacas were stratified by age such that animal age was balanced between groups. hDPP4 homozygous or heterozygous mice were balanced by genetic status across groups.
Blinding	Operators were not intentionally blinded during animal studies or sample evaluation. No explicit awareness of expected outcomes was provided.

Reporting for specific materials, systems and methods

We require information from authors about some types of materials, experimental systems and methods used in many studies. Here, indicate whether each material, system or method listed is relevant to your study. If you are not sure if a list item applies to your research, read the appropriate section before selecting a response.

Materials & experimental systems

n/a	Involved in the study
☐	☒ Antibodies
☐	☒ Eukaryotic cell lines
☒	☐ Palaeontology and archaeology
☐	☒ Animals and other organisms
☒	☐ Clinical data
☒	☐ Dual use research of concern
☒	☐ Plants

Methods

n/a	Involved in the study
☒	☐ ChIP-seq
☐	☒ Flow cytometry
☒	☐ MRI-based neuroimaging

Antibodies

Antibodies used	MERS-CoV specific primary antibodies: MERS-4, MERS-27, JC57-11, JC75-14, CDC2-A2, CDC2-C2, 4C2, G2, FIB-H1, 3A3, D12, MCA1, and Casirivimab (negative control antibody). All antibodies were produced at GenScript on a human IgG1 Fc. All Vh and Vl sequences are provided in Supplemental Table S2. Antibodies were used for BLI binding experiments at 50 µg/mL, Luminex binding at 20 µg/mL, and flow cytometry at 2.5 µg/mL.<br>Commercial antibodies:<br>goat anti-mouse IgG R-phycoerythrin secondary antibody (Southern Biotech 1030-09, used at 2 µg/mL)<br>Biotinylated anti-mouse IgG1 secondary (Southern Biotech, 1070-08, used at 4 µg/mL)<br>Biotinylated anti-mouse IgG2a secondary (Southern Biotech, 1080-08, used at 4 µg/mL)<br>anti-VSV-G (I1-mouse hybridoma supernatant, used at 1:25 dilution, CRL-2700, ATCC)<br>IFN-g monoclonal antibody (from Mabtech 3420-2A, used at 15 µg/mL)<br>IL-4 monoclonal antibody (from Mabtech 3410-2A, used at 15 µg/mL)<br>IFN-g biotin detector antibody (from Mabtech 3420-2A, used at 1 µg/mL)<br>IL-4 biotin detector antibody (from Mabtech 3410-2A, used at 1 µg/mL)<br>PE-conjugated anti-human secondary antibody (Southern Biotech 2014-09, used at 1 µg/mL)<br>Alexa Fluor 405 conjugated anti-mouse secondary antibody (Invitrogen A31553, used at 1 µg/mL)
Validation	Specific validation of antibodies was not conducted beyond qualitative assessment of expected antibody binding or activity profiles.

Eukaryotic cell lines

Policy information about cell lines and Sex and Gender in Research

Cell line source(s)	HEK293T cells (ATCC CRL-3216)<br>HeLa / DPP4 cells (Cellecta RMCOV-CDPP4HE)<br>Vero cells (ATCC CCL-81)<br>Vero-TMPRSS2 cells (Parent line: ATCC. Over-expression line produced in a collaborating lab)
Authentication	Cell lines were not authenticated
Mycoplasma contamination	Cell lines were not explicitly tested for mycoplasma contamination but were certified as mycoplasma-free upon receipt from vendors.

Commonly misidentified lines
(See ICLAC register)

N/A

Animals and other research organisms

Policy information about studies involving animals; ARRIVE guidelines recommended for reporting animal research, and Sex and Gender in Research

Laboratory animals	Mouse immunogenicity: BALB/c (Mus musculus), females 7-8 weeks of age at study start.<br>NHP immunogenicity: Cynomolgus macaques (Macaca fascicularis), males 6 years of age at study start.<br>Alpaca challenge study: Alpacas (Vicugna pacos), females 6-9 years of age at study start.<br>DPP4 transgenic mice: BALB/c (Mus musculus), females and males 122-162 days old at study start.
Wild animals	N/A
Reporting on sex	Female mice were used in mouse immunogenicity testing to reduce the likelihood of aggression between mice within immunization groups. Male NHPs were used based on animal availability. Female alpacas were used based on animal availability and due to the potentially aggressive nature of male alpacas to reduce risk in a BSL3 setting. Male and female hDPP4 transgenic mice were used and were distributed in roughly equal numbers across all immunization groups though this information was not collected during the study. Male and female animals were tested across mouse, alpaca, and NHP immunizations studies and concordance in the qualitative immune responses in these studies suggest that findings are not specific to one sex. Sex of animals in the studies conducted has been indicated in the manuscript accordingly and qualified for sex as indicated.
Field-collected samples	N/A
Ethics oversight	All animal studies were conducted in accordance with protocols approved by an Institutional Animal Care and Use Committee (IACUC). WT BALB/c mouse studies were conducted in accordance with the IACUC of Fortis Life Sciences. NHP studies were conducted at Alpha Genesis, Inc. and approved by the Committee on the Care and Use of Laboratory Animal Resources (IACUC Approval: #23-7). Alpaca and human DPP4 mouse challenge studies were approved by the CSU IACUC protocol 4926.

Note that full information on the approval of the study protocol must also be provided in the manuscript.

Plants

Seed stocks	N/A
Novel plant genotypes	N/A
Authentication	N/A

Flow Cytometry

Plots

Confirm that:

- [x] The axis labels state the marker and fluorochrome used (e.g. CD4-FITC).
- [x] The axis scales are clearly visible. Include numbers along axes only for bottom left plot of group (a 'group' is an analysis of identical markers).
- [x] All plots are contour plots with outliers or pseudocolor plots.
- [x] A numerical value for number of cells or percentage (with statistics) is provided.

Methodology

Sample preparation	Sample Preparation: HeLa cells (ATCC) were plated in a 24-well plate at a density of 2.5 x 105 cells per well in 450 µL media (RPMI + 10% FBS + 55 µM-mercaptoethanol) and allowed to adhere for 4 hours. LNPs were then diluted to 10X in PBS and added directly to cells in a volume of 50 µL. Cells were incubated with LNPs for 20 hours at which point media was removed and cells were treated with StemPro Accutase (Gibco) for 10 min. Detached cells were transferred in technical quadruplicate to a 96-well V-bottom plate and spun at 500 xg for 5 min. Cellular media was aspirated off and cells were washed once with PBS and then treated with a 1:1000 dilution of Live-or-Dye 665/685 (Biotium) in PBS for 15 min at room temperature. After live/dead staining, two wells per condition were treated with flow buffer (PBS + 1% FBS + 1 mM EDTA) for the "cell-surface

	expression" condition, while two wells per condition were fixed and permeabilized using Cytofix/Cytoperm (BD) according to the manufacturer's protocol for the "total cell expression" condition. Cells were washed with their respective buffers (flow buffer or 1X Perm/Wash (BD)), then stained with 2.5 µg/mL primary antibodies for 45 min at 4 ˚C followed by 1 µg/mL of PE-conjugated anti-human secondary antibody (Southern Biotech Cat. No. 2014-09) and Alexa Fluor 405 (AF405)-conjugated anti-mouse secondary antibody (Invitrogen Cat. No. A31553) for 45 min at 4 ˚C. Cells were then washed in their respective buffers, responded in flow buffer, and analyzed on a Cytek Aurora flow cytometer.
Instrument	Instrument: Cytek Aurora 16V-14B-8R
Software	Raw data were collected using Cytek SpectroFlo software. Data were analyzed in FlowJo v10.1 to determine the percentage of positive cells (relative to the untreated control). The percentage of positive cells was then plotted in GraphPad Prism 10.
Cell population abundance	10,000 cells per condition were analyzed in duplicate.
Gating strategy	Singlet, live, G2 and JC57-14 positive cells were determined according to the gating strategy shown in Figure S9B. Positive cells were determined relative to untreated control cells, which received PBS as opposed to the indicated LNPs.

☒ Tick this box to confirm that a figure exemplifying the gating strategy is provided in the Supplementary Information.
